# Safety and efficacy of abacavir for treating infants, children, and adolescents living with HIV: a systematic review and meta-analysis

**DOI:** 10.1016/S2352-4642(22)00213-9

**Published:** 2022-10

**Authors:** Julie Jesson, Laura Saint-Lary, Marc Harris Dassi Tchoupa Revegue, John O’Rourke, Claire L Townsend, Françoise Renaud, Martina Penazzato, Valériane Leroy

**Affiliations:** a Center for Epidemiology and Research in Population Health, Inserm U1295, Université de Toulouse, Université Paul Sabatier, Toulouse, France; b World Health Organization, Geneva, Switzerland

## Abstract

**Background:**

Abacavir is a nucleoside reverse transcriptase inhibitor recommended in paediatric HIV care. We assessed the safety and efficacy profile of abacavir used in first, second, or subsequent lines of treatment for infants, children, and adolescents living with HIV to inform 2021 WHO paediatric ART recommendations.

**Methods:**

In this systematic review and meta-analysis, we included observational and experimental studies conducted in infants aged 0–1 year, children aged 1–10 years, and adolescents aged 10–19 years living with HIV; with data on safety or efficacy, or both, of abacavir-based antiretroviral therapy (ART); published in English or French between Jan 1, 2009, and Oct 1, 2020, plus an updated search to incorporate studies published between Oct 1, 2020, and May 15, 2022. Studies could be non-randomised or non-comparative and include patients who are treatment-naive or those who previously received abacavir (only if abacavir was combined with other ART). Case studies, studies in adults aged 18 years or older, and those assessing the effect of maternal ART exposure were excluded. We extracted data related to study identifier, study design, study period, setting, population characteristics, ART treatment, and safety (any hypersensitivity reaction, death, grade 3 or 4 adverse events, treatment discontinuation, any other morbidities, and serious adverse events), and efficacy outcomes (HIV viral load and CD4 counts reported at 6 and 12 months after ART initiation). Using random-effect models, we estimated weighted pooled incidence and relative risk (RR) of outcomes. The protocol is published in PROSPERO (CRD42022309230).

**Findings:**

Of 1777 records identified, 1475 (83%) were screened after removing duplicates and a further 1421 (96%) were excluded. Of 54 full-text articles assessed for eligibility, 33 (61%) were excluded. Four records were identified from grey literature plus one duplicate from database searching, resulting in 24 studies included (two randomised controlled trials, one single-arm trial, 12 prospective cohorts, seven retrospective cohorts, and two cross-sectional studies). 19 studies described safety data and 15 described efficacy data. 18 (75%) studies were conducted in ART-naive participants. The risk of bias was considered moderate to high for most studies, and all outcomes had significant between-study heterogeneity. Data from 24 265 participants were included, of whom 7236 (30%) received abacavir. Abacavir hypersensitivity reaction was reported in nine (38%) studies, with an incidence ranging from 0·00% to 8·26% (*I*^2^=85%; p<0·0001). The incidence of death (reported in seven studies) following abacavir treatment varied from 0·00% to 5·49% (*I*^2^=58%; p=0·026). Viral suppression (<400 copies per mL) varied from 50% to 70% at 6 months (*I*^2^=92%, p<0·0001) and from 57% to 78% at 12 months (*I*^2^=88%, p<0·0001).

**Interpretation:**

Toxic effects due to abacavir use remain rare and manageable. Despite scarce data on efficacy, this meta-analysis supports the use of abacavir as a preferred first-line regimen for infants and children living with HIV.

**Funding:**

WHO.

## Introduction

In 2020, 2·8 million children and adolescents aged 19 years or younger were living with HIV worldwide, with more than 90% in sub-Saharan Africa.^1^ Despite improvements in antiretroviral therapy (ART) accessibility in resource-limited settings in the past 15 years, ART coverage remains suboptimal and a lower proportion of children receive ART (54% *vs* 74% adults).^1^

Abacavir is a nucleoside reverse transcriptase inhibitor that has progressively replaced stavudine (d4T) due to fewer toxic effects, such as lipodystrophy and metabolic abnormalities.^2^, ^3^ Abacavir is also preferred to tenofovir disoproxil fumarate for infants and children due to concerns about renal toxic effects and loss of bone mineral density in this population,^4^ and the absence of a paediatric formulation of tenofovir disoproxil fumarate.^5^ Thus, in the WHO consolidated guidelines on HIV prevention,^6^ abacavir is part of the preferred first-line regimen for children aged 4 weeks or older weighing at least 3 kg, coupled with lamivudine and dolutegravir or alternatively ritonavir-boosted lopinavir; whereas, tenofovir disoproxil fumarate is preferred to abacavir as a first-line regimen for individuals aged 10 years or older or those weighing at least 35 kg. However, abacavir use is associated with several toxic effects that need to be carefully monitored during ART initiation; a rare but concerning adverse event in children and adults is a hypersensitivity reaction, which requires immediate and permanent treatment discontinuation. Presence of the HLA-B*5701. allele—for which the frequency differs by ethnic group, with lower prevalence in Black African people than in White people^7^, ^8^—is strongly associated with the risk of an abacavir-induced hypersensitivity reaction.^9^ Abacavir is associated with increased risk of cardiovascular events in adults.^10^ In 2021, an international study^11^ estimated that the risk of cardiovascular events increased by 40% in people who received abacavir compared with those who did not receive abacavir. Although abacavir has shown similar antiviral efficacy in paediatric clinical trials to other nucleoside reverse transcriptase inhibitors,^12^, ^13^ cohort studies^14^, ^15^ in South Africa highlighted lower virological responses that need further exploration. Previous systematic reviews^16^, ^17^ assessed safety and efficacy outcomes in children and adolescents with HIV receiving abacavir and concluded that the drug was a viable option as part of first-line regimens, although only a few specific studies are available in this population. Large scale paediatric ART programmes that increase access to early diagnosis and life-long therapy are being implemented, and dolutegravir combined with abacavir is the preferred first-line regimen in children. Therefore, summarising the latest knowledge on abacavir in this population is important.

**Research in context**

**Evidence before this study**

Abacavir is a nucleoside reverse transcriptase inhibitor recommended as part of an antiretroviral first-line regimen in infants, children, and adolescents living with HIV. We searched MEDLINE, Embase, and the Cochrane Library from Jan 1, 2009, to May 15, 2022, for experimental and observational studies in English or French, reporting on the safety and efficacy outcomes associated with abacavir use in infants, children, and adolescents living with HIV. Previous systematic reviews and meta-analyses in 2015 and 2016 assessed safety and efficacy of abacavir use in this study population. Since then, the number of eligible studies has substantially increased, with new findings from two randomised controlled trials and eight prospective cohorts (including two conference abstracts). Universal treatment for all patients living with HIV started to be recommended in 2015, and with the implementation of a large-scale paediatric ART programme, the state of knowledge on this drug needs to be updated.

**Added value of this study**

This systematic review and meta-analysis combines safety and efficacy outcomes to bring a comprehensive overview of abacavir use in infants, children, and adolescents living with HIV, with a focus on the past decade. Our results confirmed that abacavir toxicity remains rare and manageable in this population. Abacavir efficacy compared with other drugs remains unclear, with lower efficacy observed in some cohorts than seen in randomised clinical trials. These findings should be interpreted with caution because the results are highly heterogenous between the included studies.

**Implications of all the available evidence**

Our results confirmed that abacavir can be safely used among infants and children, especially when ART initiation is combined with close monitoring within the first months to prevent abacavir-induced hypersensitivity reaction. However, data remain insufficient for adolescents. Adverse events, not directly attributable to abacavir, remained common in children initiating ART. The introduction of new paediatric formulations in the past few years might reduce the incidence of adverse events and improve efficacy, which needs to be systematically reported and assessed to strengthen the current evidence.

We aimed to assess the safety and efficacy profile of abacavir used in first, second, or subsequent lines of treatment for infants, children, and adolescents living with HIV to inform 2021 WHO paediatric ART recommendations.

## Methods

### Search strategy and selection criteria

In this systematic review and meta-analysis, we included observational and experimental studies conducted in infants aged 0–1 year, children aged 1–10 years, and adolescents aged 10–19 years living with HIV; with data on safety or efficacy, or both, of abacavir-based ART; published in English or French between Jan 1, 2009, and Oct 1, 2020. An updated search strategy was also conducted to incorporate studies published between Oct 1, 2020, and May 15, 2022. Studies could be non-randomised or non-comparative and the study population could consist of patients who are treatment-naive or those who previously received abacavir (only if abacavir was combined with other ART). Case studies, studies in adults aged 18 years or older, and those assessing the effect of maternal ART exposure were excluded.

We searched MEDLINE (via the Web of Science), Embase, and the Cochrane Library using free text and index terms combining HIV, abacavir, children, and adolescents (appendix pp 1–4). We searched grey literature sources for patient-level data or summary estimates by hand screening reference lists of systematic reviews, international guidelines on HIV treatment, clinical trial registries, and targeted conference abstracts published between January, 2018 and March, 2022 (appendix p 5). Study authors were contacted when further clarification was needed.

Titles, abstracts, and the full text were independently screened by two reviewers (JJ and VL), with technical support using the Rayyan web app.^18^ Main reasons for exclusion during the full text review stage were documented (appendix pp 6–8). Discordance was resolved by discussions with the project team. This systematic review and meta-analysis is an update of a previous review^16^ published in 2016 regarding the safety outcomes, and here, we include new results on efficacy outcomes. The protocol is published in PROSPERO (CRD42022309230).

### Data analysis

We extracted data related to study identifier, study design, study period, setting, population characteristics (sex and age groups), ART treatment (comparative groups and whether patients previously received ART treatment), and safety and efficacy outcomes (using a piloted data extraction spreadsheet). Data extraction was done by a single unmasked reviewer (JJ) and checked and validated independently by a second reviewer (VL). Endnote software (version X9.2) and the Rayyan web app were used to identify and exclude duplicate data. Reported safety outcomes were any hypersensitivity reaction, death, grade 3 or 4 adverse events, treatment discontinuation, any other morbidities, and serious adverse events (defined as any life-threatening adverse event or reaction that requires hospitalisation, results in persistent or substantial disability or incapacity, or any other important medical condition). Efficacy outcomes extracted were HIV viral load (expressed as virological suppression using a threshold of 400 or 50 copies per mL or as other viral load outcomes if not expressed as a threshold) and CD4 counts (expressed in cell counts or percentages); both commonly reported at 6 and 12 months after ART initiation.

The quality of scientific research and overall risk of bias were assessed using the Cochrane risk of bias tool (version 2.0) for randomised controlled trials (RCTs), the 2013 US National Institutes of Health quality assessment tool for non-randomised interventional studies, and Clarity's clinical advances using the research and information translation tool for observational studies.

Safety and efficacy outcomes according to abacavir exposure were first described within a narrative synthesis and pooled incidences were then estimated with 95% CIs using a meta-analysis with a random-effect model and building forest plots. Heterogeneity between studies was assessed by quantifying the inconsistency between incidence rate estimates with Q, χ^2^, and *I*^2^ tests calculated using MetaXL software (version 5.3; EpiGear International, Sunrise Beach, QL, Australia). Only analyses with an *I*^2^ of 90% or less are displayed using forest plots. Relative risk of safety and efficacy outcomes between the abacavir-containing regimen group (intervention group) and non-abacavir-containing regimen group (control group) were summarised by risk ratio (RR) and 95% CI using DerSimonian and Laird random-effect models and building forest plots. Heterogeneity between risk ratio was assessed using the τ^2^ statistic and calculated with Review Manager (version 5.4; Cochrane Collaboration, Copenhagen, Denmark).

### Role of the funding source

The funder of the study had no role in study design, data collection, data analysis, data interpretation, or writing of the report.

## Results

**Figure 1:**
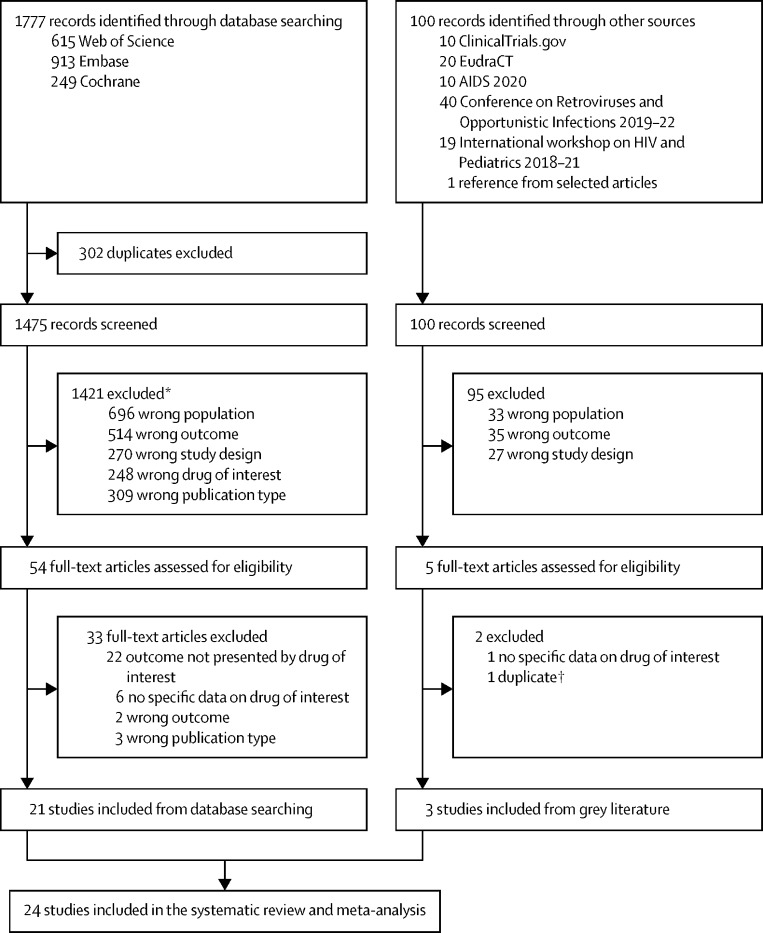
Study selection *Reasons for exclusion were not mutually exclusive. †One article was identified using database searching and other sources (AIDS 2020 published abstract).^19^

Of 1777 records identified through database searching, 1475 (83%) were screened after removing duplicates and a further 1421 (96%) were excluded after title and abstract screening (figure 1). Of 54 full-text articles assessed for eligibility, 33 (61%) were excluded. Three records were identified from grey literature plus one duplicate from database searching and other sources (AIDS 2020 published abstract),^19^ resulting in a total of 24 studies included in the systematic review and meta-analysis.

Data from 24 265 participants were included, of whom 7236 (30%) received abacavir. 19 (79%) of 24 studies included safety data and 15 (63%) included efficacy data. Two (8%) studies were comparative RCTs^13^, ^20^ and one (4%) was a single-arm, phase 2 trial^21^ with abacavir used as backbone. 12 (50%) were prospective cohort studies,^15^, ^22^, ^23^, ^24^, ^25^, ^26^, ^27^, ^28^, ^29^, ^30^, ^31^, ^32^ including one cohort nested in an RCT.^22^ Seven (29%) were retrospective cohort studies^14^, ^33^, ^34^, ^35^, ^36^, ^37^, ^38^ and two (8%) were cross-sectional surveys.^19^, ^39^ These studies were conducted in various settings; nine (38%) in southern Africa (Zambia, South Africa, and Zimbabwe), six (25%) in eastern Africa (Ethiopia, Uganda, and Malawi), two (8%) in western Africa (Ghana and Nigeria), three (13%) in south Asia (India), three (13%) in Europe, and three (13%) in North America. Four (17%) studies were multiregional.^13^, ^21^, ^28^, ^29^ The study period ranged from 1998 to 2019, and eleven (46%) studies started in 2010 or later. 18 (75%) studies were conducted in ART-naive participants. Follow-up varied from 48 weeks to more than 5 years. Median age ranged from 32 days (IQR 6–66) to 17 years (IQR 16–18) at inclusion. Six (25%) studies included infants younger than 12 months and eight (33%) included adolescents aged 10 years or older at baseline (table 1).

**Table 1:** Characteristics of included studies on abacavir safety and efficacy

	**Country**	**Study period**	**Study design and follow-up**	**Patients receiving abacavir**	**ART regimen of comparison group**	**Previous ART treatment and duration**	**Median or mean patient age at baseline**	**Breakdown by sex**
Mulenga et al (2016)^13^	Zambia and Uganda	2010–11	Open-label, parallel-group, multi-arm trial (CHAPAS-3); 96 weeks	164 (34%) of 478	Stavudine (n=156) versus zidovudine (n=158) versus abacavir; backbone of lamivudine and either nevirapine or efavirenz	ART naive (76%) versus previously received ART (24%); stavudine for >2 years with viral load <50 copies per mL	Median age 2·6 years (IQR 1·6–4·0) for ART-naive patients versus 6·2 years (5·5–7·2) for those who previously received ART	49% male and 51% female ART naive; 52% male and 48% female previously received ART
Dirajlal-Fargo et al (2017)^22^	Uganda	2010–11	Sub-study nested within the CHAPAS-3 trial; 48 weeks	41 (35%) of 118	Stavudine (n=42) versus zidovudine (n=35) versus abacavir; backbone of lamivudine and either nevirapine or efavirenz	ART naive	Median age 2·8 years (IQR 1·7–4·3)	49% male and 51% female
Strehlau et al (2018)^20^	South Africa	2010–13	Unblinded open-label RCT; 56 weeks (in children with viral suppression without lipodystrophy)	107 (50%) of 213 substituted from stavudine to abacavir	106 (50%) remained on stavudine	Previously received ART (mean duration 3·5 years [SD 0·26] for abacavir *vs* 3·3 years [0·8] for stavudine)	Mean age 4·2 years (SD 1·0) for patients receiving abacavir versus 4·2 years (0·9) for those receiving stavudine	47% male and 53% female
Fortuny et al (2014)^21^	North America, Europe, and South Africa	2004–10	Phase 2, open-label, multicentre, single-arm trial; 48 weeks	109 (100%) of 109	Fosamprenavir, with ritonavir (n=70) or without ritonavir (n=39); abacavir used as backbone; no comparison group	ART naive (38%), previously received ART and were protease inhibitor-naïve (26%), previously received ART and protease inhibitor (37%)	Median age 9 years (IQR 2–18)	47% male and 53% female
Technau et al (2014)^15^	South Africa	1998–2013	Prospective cohort; 15 months	1536 (16%) of 9543	Stavudine (n=8007) versus abacavir, backbone of lamivudine and either efavirenz or ritonavir-boosted lopinavir	ART naive	Median age 7 months (IQR 4–18) for patients receiving ritonavir-boosted (lopinavir based) and 96 months (63–129; efavirenz based)	50% male and 50% female
Fortuin-de Smidt et al (2017)^23^	South Africa	2004–14	Prospective cohort; median 41·0 months (IQR 14–72)	1043 (29%) of 3579	Lamivudine–stavudine plus either efavirenz (most common regimen used) in children younger than 3 years (n=1199) or ritonavir-boosted lopinavir in those older than 3 years (n=842)	ART naive	Median age 44 months (IQR 13–89)	50% male and 50% female
de Waal et al (2020)^24^	South Africa	2006–17	Prospective cohort; median 15·0 months (IQR 4·2–36·2) for abacavir and 10·6 months (IQR 2·5–25·6) for zidovudine 10·6	931 (73%) of 1275	Zidovudine	ART naive	Median age 67 days (IQR 48–80) for abacavir and 32 days (6–66) for zidovudine	44% male and 56% female
Patel et al (2012)^25^	USA	1993–2007	Prospective cohort; median 5·5 years	789 (26%) of 3035	Abacavir versus no abacavir	ART naive (17%) versus previously received ART (83%)	44% of patients aged 5 years or younger	49% male and 51% female
Patel et al (2014)^26^	USA	2007–09	Prospective cohort; 4 years	46 (28%) of 165	Abacavir versus no abacavir	Previously received ART (median duration 11·0 years [IQR 7·7–12·5]) *vs* 0·0 years [0·0–3·3] for abacavir)	Median age 16·7 years (IQR 15·9–17·8)	49% male and 51% female
Tadesse et al (2019)^27^	Ethiopia	2017–19	Prospective cohort; 48 weeks	37 (33%) of 111	Lamivudine–efavirenz plus either zidovudine (n=15), abacavir (n=37), or tenofovir disoproxil fumarate (n=59)	ART naive	Median age 9 years (IQR 5–12)	47% male and 53% female
Nahirya-Ntege et al (2011)^28^	Uganda and Zimbabwe	2007–10	Prospective cohort, nested within the ARROW trial; range 3·5–5·0 years	1150 (100%) of 1150	All children received ABC and lamivudine plus nevirapine or efavirenz and those in the induction-maintenance groups also received zidovudine	ART naive	Age ranged from 3 months to 17 years (median 6 years)	50% male and 50% female
Crichton et al (2020)^29^	Europe (11 countries)	2000–16	Prospective cohort; median 4·6 years (IQR 1·5–9·7; outcomes at 12 months)	139 (100%) of 139	No comparison group; abacavir-lamivudine plus either ritonavir-boosted lopinavir (n=54), zidovudine and nevirapine (n=45), or nevirapine (n=19); other (n=21)	ART naive	Median age 62 days (IQR 35–78)	40% male and 60% female
Pareek et al (2019)^30^	India	2015–16	Prospective cohort; 12 months	48 (100%) of 48	No comparison group; abacavir with lamivudine plus nevirapine (n=32) or abacavir with lamivudine plus efavirenz (n=16)	ART naive	Mean age 9·8 years (SD 3·4)	75% male and 25% female
Manglaniet al (2018)^31^	India	2013–14	Prospective cohort; 12 months (study period)	100 (100%) of 100	No comparison group	Abacavir taken for a median of 7 days (range 3–15) in those who were to receive ABC (n=90) and 88 days (16 −774) in those who were receiving ABC (n=10)	Median age 11 years (range 2–18)	61% male and 39% female
Chakravarty et al (2016)^32^	India	2013–14	Prospective cohort; 12 months	101 (100%) of 101	No comparison group	ART naive (n=27) or previously received ART (n=73; median duration 12·5 months [IQR 0·0–24·0])	Mean age 5·8 years (SD 3·5)	70% male and 30% female
Cassim et al (2017)^33^	South Africa	2005–13	Retrospective case-control; 12 months	57 (33%) of 171	Stavudine versus abacavir; backbone of lamivudine and ritonavir-boosted lopinavir	ART naive	Median age 3·11 months (IQR 1·98–6·05)	43% male and 57% female
Technau et al (2013)^14^	South Africa	2004–11	Retrospective cohort; 12 months	402 (20%) of 2036	Stavudine (n=1634) versus abacavir; backbone of lamivudine plus either efavirenz (n=962 in the stavudine group *vs* n=210 in the abacavir group) or ritonavir-boosted lopinavir (n=672 *vs* n=192)	ART naive	Median age 10 months (IQR 4–20) for ritonavir-boosted (lopinavir based) and 86 months (60–119; efavirenz based)	50% male and 50% female
Frange et al (2011)^34^	France	2000–09	Retrospective cohort; median 36 months (IQR 18–72) during ritonavir-boosted lopinavir treatment	19 (44%) of 43	Ritonavir-boosted lopinavir with backbone lamivudine (n=36), zidovudine (n=26), stavudine (n=5), or abacavir (n=19)	ART naive	Median age 4·8 years (IQR 1·8–8·0)	60% male and 40% female
Langs-Barlow et al (2013)^35^	Ghana	2004–11	Retrospective cohort; no follow-up information	44 (11%) of 403	Comparison group (abacavir exposure yes or no); most common regimen of zidovudine (n=327) plus lamivudine (n=331) and either efavirenz (n=250) or nevirapine (n=93)	Both ART naive (8%) and previously received ART (82%)	Mean age 108·1 months (SD 41·4) for ART naïve patients and 97·3 months (40·7) for those who previously received ART	44% male and 56% female ART naive; 52% male and 48% female previously received ART
Mega et al (2020)^36^	Ethiopia	2014–17	Retrospective cohort; 42 months	87 (49%) of 179	Zidovudine plus lamivudine and either efavirenz or ritonavir-boosted lopinavir or nevirapine	Previously received ART for at least 6 months	Mean age 6·53 years (SD 2·83)	45% male and 55% female
Mega et al (2020)^37^	Ethiopia	2015–17	Retrospective cohort; 42 months	87 (49%) of 179	Zidovudine plus lamivudine and either efavirenz or ritonavir-boosted lopinavir or nevirapine	Previously received ART for at least 6 months	Mean age 6·53 years (SD 2·83)	45% male and 55% female
Oshikoya et al (2012)^38^	Nigeria	2008–10	Retrospective cohort; 42 months	31 (39%) of 80	At enrolment: zidovudine plus nevirapine (n=74), efavirenz (n=5), or abacavir and ritonavir-boosted lopinavir (n=1); change in ART regimen (n=33, including n=31 with abacavir)	ART naive	Median 3·0 years (IQR 1·1–6·0)	43% male and 57% female
Natukunda et al (2017)^39^	South Africa	2014–15	Cross-sectional	231 (46%) of 501	Several different regimen including abacavir, lamivuding, and efavirenz (n=165) or tenofovir disoproxil fumarate, emtricitabine, and efavirenz (n=116)	Previously received ART for 5 years (IQR 2–10)	Median age 14 years (IQR 12–16)	46% male and 54% female
Ahimbisibwe et al (2020)^19^	Malawi	2018–19	Cross-sectional	32 (4%) of 806	No information on regimen; nucleoside reverse transcriptase inhibitor zidovudine (n=630) or tenofovir disoproxil fumarate (n=142) plus efavirenz, ritonavir-boosted lopinavir, or nevirapine	Previously received ART for at least 6 months	Median age 10 years (IQR 7–13)	53% male and 47% female

ART=antiretroviral therapy. RCT=randomised controlled trial.

The two comparative RCTs were classified as low risk of bias, whereas the single-arm trial was classified as moderate risk. Of 21 observational studies, one (5%) was classified as low risk of bias and eight (40%) as moderate or unclear risk of bias. 12 (60%) were classified as high risk mainly because of the retrospective study design, which reduces confidence in exposure and outcome assessments (appendix pp 9–10).

**Figure 2:**
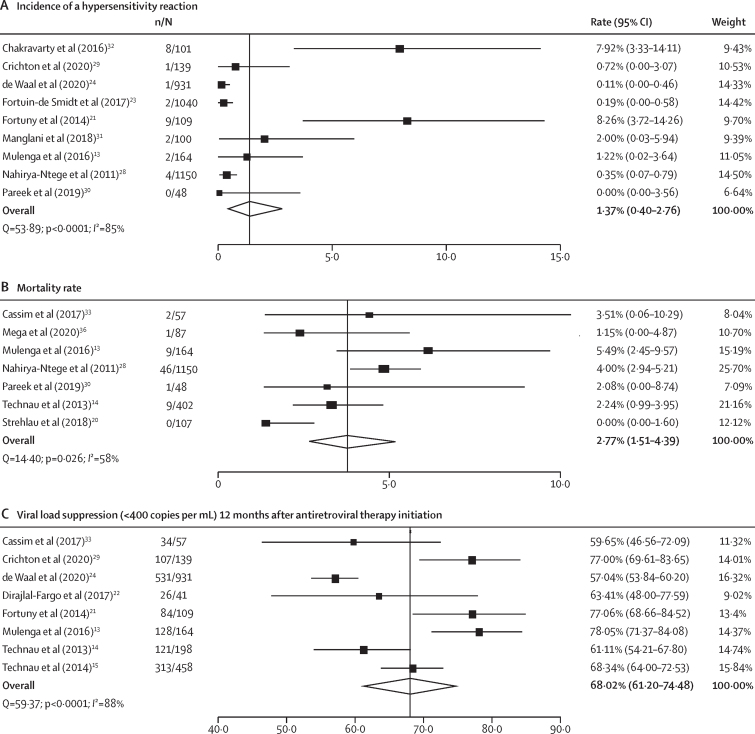
Cumulative incidence for safety and efficacy outcomes Only analyses with an *I*^2^ of 90% or less are displayed. Safety outcomes shown as the incidence of a hypersensitivity reaction (A) and mortality rate (B) at 12 months. Efficacy outcomes shown as viral load suppression (C) at 12 months.

Regarding the safety outcomes (table 2), abacavir hypersensitivity reaction was reported in nine studies (38%; one RCT, one single-arm trial, and seven prospective cohorts)^13^, ^21^, ^23^, ^28^, ^29^, ^30^, ^31^, ^32^ with an incidence ranging from 0·00% to 8·26% and a significant between-study heterogeneity (*I*^2^=85%; p<0·0001; figure 2A). The RCT by Mulenga and colleagues^13^ compared hypersensitivity reactions by drug regimen and reported an incidence of two (1%) of 164 for the abacavir group, five (3%) of 156 for the stavudine group, and one (1%) of 158 for the zidovudine group with no statistical difference between groups (p=0·21). All children receiving abacavir with grade 1–4 hypersensitivity reactions stopped the drug without any further adverse effects reported. Of the nine (8%) of 109 hypersensitivity reactions in the single-arm trial by Fortuny and colleagues,^21^ two were related to fosamprenavir or ritonavir according to the investigators and two occurred after abacavir was stopped and were related to cotrimoxazole, and no deaths were reported. In the prospective cohort study by Chakravarty and colleagues,^32^ eight (8%) of 101 children who received abacavir developed a clinically diagnosed hypersensitivity reaction and symptoms resolved after stopping abacavir in all children. Of these eight children, four with concomitant illness were HLA-B05701 negative and two carried the HLA-B05701 allele. The other six studies reported low rates of hypersensitivity reactions (all lower than 2%).

**Table 2:** Safety outcomes in children and adolescents receiving an abacavir-containing regimen

	**Age group**	**Control group**	**Hypersensitivity reactions**	**Grade 3 or 4 adverse events**	**Mortality**	**Treatment discontinuation**	**Morbidities and adverse events**
Mulenga et al (2016)^13^	Children	Stavudine and zidovudine	Abacavir (two [1%]) versus stavudine (five [3%]) and zidovudine (one [1%])	Abacavir (51 [31%]) versus stavudine (46 [29%]) and zidovudine (53 [34%])	Abacavir (nine [5%]) versus stavudine (seven [4%]) and zidovudine (three [2%])	NA	Primary endpoint (ie, grade 2 or greater clinical adverse event or confirmed grade 3 or grade 4 laboratory adverse event; 64% with abacavir *vs* 67% with stavudine *vs* 65% with zidovudine), lipodystrophy (0% *vs* 1% *vs* 0%), and mitochondrial disease (1% *vs* 1% *vs* 0%)
Dirajlal-Fargo et al (2017)^22^	Children	Stavudine and zidovudine	NA	NA	NA	NA	Median change in Homeostatic model assessment of insulin resistance at 48 weeks (6% [IQR −34% to 124%] with abacavir *vs* 14% [−29% to 97%] with stavudine *vs* −1% [−30% to 69%] with zidovudine)
Strehlau et al (2018)^20^	Children	Stavudine	NA	NA	0	NA	Lipodystrophy (five [5%] with abacavir *vs* 17 [16%] with stavudine), mean weight-for-age Z score (−0·72 [SD 1·0] *vs* −0·72 [1·0]; p=0·96), and mean height-for-age Z score (−1·21 [SD 1·0] *vs* −1·18 [1·0]; p=0·85)
Fortuny et al (2014)^21^	Children and adolescents	NA	Abacavir (nine [8%])	Abacavir (22 [32%]	NA	Abacavir (four [4%] of 109 patients)	At least one adverse event (42 [39%] with abacavir)
Fortuin-de Smidt et al (2017)^23^	Children and adolescents	Lamivudine and stavudine plus efavirenz or ritonavir-boosted lopinavir (most common)	Abacavir (two [<1%] *vs* not reported for control)	NA	NA	Abacavir (58, 30 cases [95% CI 23–39] per 1000 patient-years) versus control (841, 87 cases [81–93] per 1000 patient-years)	Treatment-limiting toxicity (three, 1·6 cases [95% CI 0·5–4·8] per 1000 patient–years with abacavir *vs* 46, 50·6 cases [46·2–55·4] per 1000 patient-years with control; adjusted HR 30·8 [95% CI 4·3–220·2])
de Waal et al (2020)^24^	Infants	Zidovudine	Abacavir (one [<1%]) *vs* not reported for control)	NA	NA	12-month treatment discontinuation; abacavir (61 [8%] of 789) versus control (HR 0·16 [95% CI 0·10–0·23])	NA
Patel et al (2012)^25^	Children	Abacavir versus no abacavir	NA	NA	NA	NA	Incident cardiomyopathy (eight [8%] with abacavir *vs* 91 [92%] with no abacavir; adjusted OR 0·7 [95% CI 0·3–1·5])
Patel et al (2014)^26^	Adolescents	Abacavir versus no abacavir	NA	NA	NA	NA	Abdominal aorta: Pathobiological Determinants of Atherosclerosis in Youth score of 0 (29 [23%]) or ≥1 (17 [44%]), with current use of abacavir (adjusted OR 1·8 [95% CI 0·6–5·3])
Nahirya-Ntege et al (2011)^28^	Infants, children, and adolescents	NA	Abacavir (four [<1%])	NA	Abacavir (46 [4%])	Abacavir (seven [13%] of 52)	Serious adverse events (52 [5%] with abacavir; 40 occurred within the first 4 weeks of ART)
Crichton et al (2020)^29^	Infants	NA	Abacavir (one [<1%])	Abacavir (eight [6%] within the first 7 days of ART)	NA	Due to ART safety (four of 139; cumulative incidence 3·6% [95% CI 1·4–7·8]) and for any reason (15 of 139; cumulative incidence 11·8% [7·3–18·9]) at 12 months with abacavir	NA
Pareek et al (2019)^30^	Children and adolescents	NA	Abacavir (none [0%])	NA	Abacavir (one [2%] not related to abacavir hypersensitivity reaction)	NA	Abacavir side-effects included fever (eight [16%]); skin rash (seven [14%]); respiratory (six [12%]), gastrointestinal (two [4%]), and constitutional (one [2%]) symptoms
Manglani et al (2018)^31^	Children and adolescents	NA	Abacavir (two [2%]; both HLA-B*5701 positive)	NA	NA	NA	NA
Chakravarty et al (2016)^32^	Children	NA	Abacavir (eight [8%]; two HLA-B*5701 positive)	NA	NA	NA	One had a febrile illness, two had skin infections, and two had concomitant pulmonary tuberculosis; all symptoms resolved after stopping abacavir
Cassim et al (2017)^33^	Infants	Stavudine	NA	NA	Abacavir (two [4%]) versus control (nine [8%])	NA	Median weight-for-age Z score at 6 months (−0·93 [IQR −1·42 to 0·03] with abacavir *vs* −1·18 [−1·95 to 0·03] with control; p=0·18) and at 12 months (−0·70 [−1·25 to 0·17] *vs* −0·64 [−1·44 to 0·22]; p=0·93), and median height-for-age Z score at 6 months (−1·65 [−2·41 to −0·67] with abacavir *vs* −1·58 [−2·65 to −0·74] with control; p=0·67) and at 12 months (−1·91 [−2·64 to −1·23] *vs* −1·72 [−2·41 to −0·73]; p=0·25)
Technau et al (2013)^14^	Infants and children	Stavudine with efavirenz or ritonavir-boosted lopinavir	NA	NA	Abacavir group with ritonavir-boosted lopinavir (six [3%]) or efavirenz (three [1%]); control group with ritonavir-boosted lopinavir (24 [4%]) or efavirenz (25 [3%])	NA	NA
Langs-Barlow et al (2013)^35^	Children	Zidovudine–lamivudine plus either efavirenz or nevirapine (most common)	NA	NA	NA	NA	Increased risk of positive Enquête Périnatale Française score for mitochondrial toxic effects due to abacavir exposure (OR 4·76 [95% CI 2·39–9·43])
Mega et al (2020)^36^	Children	Zidovudine, with efavirenz or ritonavir-boosted lopinavir or nevirapine	NA	NA	Abacavir (one [1%]; median survival time 273 days [IQR 123–569]) versus control (three [3%]; median survival time 366 days [86–676])	NA	Opportunistic infections (29 [33%] of 87 with abacavir; incidence of 8·8 per 100 000 person-years *vs* 29 [32%] of 92 with control; incidence 6·9 per 100 000 person–years; incidence rate ratio 0·87 [95% CI 0·49–1·53]; p=0·30) and pneumonia (16 [18%] *vs* 15 [16%])
Oshikoya et al (2012)^38^	Children	Zidovudine–lamivudine plus either nevirapine, efavirenz, or abacavir-ritonavir-boosted lopinavir	NA	NA	NA	NA	Skin rash events (two [2%] of 93 for abacavir *vs* 91 [98%] of 93 for control); gastrointestinal, including vomiting, nausea, diarrhoea, and abdominal pain (16 [17%] *vs* 13 [14%]); pallor (one [1%] *vs* 11 [12%]); and headache (six [6%] *vs* not reported)
Natukunda et al (2017)^39^	Adolescents	Several; abacavir–lamivudine–efavirenz (33%) or tenofovir disoproxil fumarate– emtricitabine–efavirenz (23%)	NA	NA	NA	NA	Adjusted according to abacavir-containing regimens (yes *vs* vo); ≥3 self-reported symptoms (OR 0·94 [95% CI 0·53–1·67]), skin rash (0·65 [0·40–1·05]), diarrhoea (0·62 [0·38–1·01]), nausea or vomiting (0·99 [0·61–1·61]), and stomach problems (0·98 [0·60–1·60])

Age groups (infants aged 0–12 months, children aged 1–10 years, and adolescents aged 10–19 years). ART=antiretroviral therapy. HR=hazard ratio. NA=not applicable. OR=odds ratio.

All-cause treatment discontinuation was reported in one RCT^21^ and four prospective cohorts,^23^, ^24^, ^28^, ^29^ with an incidence ranging from 1% to 14% and significant between-study heterogeneity (*I*^2^=97%; p<0·001; table 2).

Five studies^13^, ^21^, ^28^, ^29^, ^36^ described adverse events such as skin rash, pneumonia, or gastrointestinal symptoms. Two studies^13^, ^21^ specifically reported grade 3 or 4 adverse events. In the RCT by Mulenga and colleagues,^13^ 917 (835 clinical and 40 laboratory) grade 2–4 clinical or grade 3–4 laboratory adverse events occurred in 104 (67%) of 156 children receiving stavudine, 103 (65%) of 158 receiving zidovudine, and 105 (64%) of 164 receiving abacavir (hazard ratio [HR] 0·99 [95% CI 0·75–1·29]; p=0·63 for zidovudine *vs* stavudine; HR 0·88 [0·67–1·15] for abacavir *vs* stavudine). Two prospective studies by Patel and colleagues explored cardiovascular risks in children^25^ and adolescents,^26^ and found no effects of abacavir use on incident cardiomyopathy (adjusted odds ratio 0·7 [95% CI 0·3–1·5]). Other adverse events were lipodystrophy,^20^ mitochondrial toxic effects,^13^, ^35^ change in insulin resistance,^22^ and growth outcomes (table 2).^20^, ^33^

**Figure 3:**
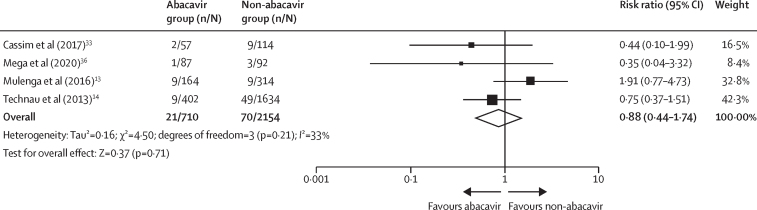
Relative risk ratio of mortality rate in children and adolescents who received abacavir-containing regimen compared with non-abacavir-containing regimen Only analyses with an *I*^2^ of 90% or less are displayed.

Mortality rate was reported in seven studies.^13^, ^14^, ^20^, ^28^, ^30^, ^33^, ^36^ The incidence of death following abacavir treatment varied from 0·00% to 5·49% with significant between-study heterogeneity (*I*^2^=58%; p=0·026; figure 2B). Four studies compared mortality rate with ART, showing a homogeneous pooled estimate for children receiving abacavir versus those receiving stavudine or zidovudine regimens (relative risk [RR] of death 0·88 [95% CI 0·44–1·74]; τ^2^=16%; test for the overall effect p=0·71; figure 3). The overall mortality rate was 2·77% (95% CI 1·51–4·39; p=0·026) and viral load suppression rate was 68·02% (61·20–74·48; p<0·0001; figure 2).

Regarding efficacy outcomes, five studies reported viral load suppression at a threshold of 400 copies per mL at 6 months,^14^, ^15^, ^24^, ^29^, ^33^ and eight studies at 12 months (appendix pp 11–13).^13^, ^14^, ^15^, ^21^, ^22^, ^24^, ^29^, ^33^ Viral suppression varied from 49·94% to 70·00% at 6 months and from 57·04% to 78·05% at 12 months, with significant heterogeneity between studies (*I*^2^=92% for the 6-month data and *I*^2^=88% for the 12-month data; p<0·0001; figure 2C). In a large prospective cohort in 2014 by Technau and colleagues,^15^ a significantly lower viral suppression rate was reported in the abacavir group than the stavudine group at 6 months (RR 0·56 [95% CI 0·43–0·72] for efavirenz and 0·49 [0·40–0·60] for ritonavir-boosted lopinavir; p<0·0001) and 12 months, regardless of the backbone therapy (RR 0·56 [0·36–0·86] for efavirenz and 0·52 [0·39–0·69] for ritonavir-boosted lopinavir; p<0·0001; appendix pp 11–14). Similar results were reported in a retrospective cohort in 2013, by the same authors.^14^ Whereas in the retrospective case–control study by Cassim and colleagues,^33^ viral suppression rates were similar between the abacavir and stavudine groups at 6 (p=0·13) and 12 months (p=0·53).

Four studies reported CD4 data related to abacavir treatment,^20^, ^30^, ^33^, ^37^ and three of these studies compared abacavir with other ARTs. In the studies by Strehlau and colleagues^20^ and Cassim and colleagues,^33^ the CD4 percentages (defined as the proportion of all lymphocytes that are CD4 cells) were similar between the abacavir and stavudine groups over time (at 26, 32, 52, and 56 weeks; appendix p 14). In the study by Mega and colleagues,^37^ CD4 cell counts after 6 months were significantly higher in the zidovudine group than the abacavir group (p=0·004).

## Discussion

This systematic review and meta-analysis showed that overall, abacavir can be used safely and effectively for infants and children, whereas specific data on adolescents remain insufficient, which aligns with the 2021 WHO paediatric ART recommendations.^40^ The major abacavir-related toxic effect, hypersensitivity reaction, was reported in less than 2% of participants for most (seven [78%] of nine studies). Other adverse events reported were not specifically associated with abacavir use. There was no difference in the risk of mortality for participants receiving abacavir compared with those receiving other antiretroviral drugs. Except in two South African cohort studies that highlighted a lower viral suppression rate in the abacavir group,^14^, ^15^ CD4 counts and viral load at 6 or 12 months after ART initiation were not different between abacavir and stavudine-based regimens. One study showed higher CD4 count gain at 6 months for zidovudine-based regimens compared with abacavir-based regimens.^37^ Most outcomes were reported heterogeneously between studies, and except for two randomised trials, studies were considered of moderate to high risk of bias. Therefore, data interpretation needs to be made cautiously.

Hypersensitivity reaction is the main toxic effect associated with abacavir use. In this systematic review and meta-analysis, no hypersensitivity reaction cases led to worse adverse events, such as death, and symptoms rapidly resolved after abacavir discontinuation. The incidence of hypersensitivity reactions ranged from 0% to 2% in seven (78%) of nine studies which reported these reactions, and all resolved with abacavir cessation. The other two studies reported a hypersensitivity reaction incidence of 8%.^21^, ^32^ The first was a prospective cohort study^32^ conducted between 2013 and 2014 in India, with a specific aim to observe the incidence of clinically diagnosed hypersensitivity reaction. This study was at high risk of bias due to a non-specific definition of the outcome, which could have led to overestimating the incidence of abacavir-induced hypersensitivity reaction. The second study was a multiregional open-label RCT^21^ conducted between 2004 and 2010, evaluating the pharmacokinetics and safety of fosamprenavir-based regimens, with abacavir used as a backbone. No data on HLA-B05701 screening before ART initiation were available in this study, which might partly explain the high rate of an abacavir-induced hypersensitivity reaction. Screening of HLA-B*5701 is now recommended before initiating abacavir-based regimens in all people living with HIV to prevent the onset of a hypersensitivity reaction. Therefore, the low hypersensitivity reaction incidence reported in other studies might be explained by the common use of HLA-B*5701 screening before ART initiation. This screening might be more easily implemented in HIV clinics participating in research programmes, explaining why the incidence of hypersensitivity reaction reported in scientific publications is low. However, the test for HLA-B*5701 is costly and not done routinely in HIV clinics within resource-limited settings, which need to be further supported to detect and reduce any abacavir-induced hypersensitivity reaction. Although testing for HLA-B*5701 is a standard-of-care in high-income countries, gaps in terms of cost and access remain in low-income and middle-income countries, which could be addressed by developing rapid and inexpensive tests.^41^, ^42^

Two studies conducted by Patel and colleagues^25^, ^26^ did not find an increased risk of cardiovascular disease associated with abacavir use in children and adolescents living with HIV. However, in the study in adolescents, the median duration of abacavir use was less than 1 year, which might be too short in terms of drug exposure to observe cardiovascular adverse events. Several observational studies conducted in adults^10^, ^43^ highlighted higher risk of cardiovascular disease for those using abacavir, whereas a pooled analysis of findings in adults enrolled in clinical trials showed no difference in the risk associated with abacavir use.^44^ Further studies assessing the causes of cardiovascular disease in children and adolescents receiving long-term ART are needed to better prevent comorbidities, especially in resource-limited settings in which most of this population live and where monitoring tools are scarce.^45^

This systematic review and meta-analysis only found a few eligible studies published between 2009 and 2022, and data collection across these studies was not homogeneous or standardised. Few studies specifically addressed abacavir safety and efficacy in adolescents, which does not allow abacavir to be recommended for this age group. Few participants initiated ART in infancy, and two of seven articles focusing on infants were conference abstracts.^24^, ^29^ Most similar studies were comparing abacavir use with stavudine use, which is no longer recommended for children as a first-line regimen. None of the studies described abacavir safety and efficacy when combined with antiretrovirals introduced in the past few years, such as integrase strand transfer inhibitors, whereas dolutegravir-based regimen could lead to lower viral suppression in children with predicted abacavir resistance as found in a recent study conducted in Kenya.^46^ Questions remain around drug susceptibility for abacavir when used as second-line ART. Only the CHAPAS-3 trial^13^ identified a lower susceptibility to abacavir as a second-line regimen for virologically suppressed children receiving first-line zidovudine. This lower susceptibility could affect virological success and decrease CD4 T-cell count. No data reported on children receiving second-line ART due to unsuccessful treatment. Further research reporting results by ART rather than the regimen are needed to better address such crucial questions (eg, the efficacy of nucleoside reverse transcriptase inhibitor backbone combined with integrase strand transfer inhibitors and ART use as second-line treatment, especially in the context of unsuccessful treatment). Similarly, growth outcomes such as weight-for-age and height-for-age might be associated with the type of ART; however, few studies describe this association with conflicting results when comparing protease inhibitor-based with non-nucleoside reverse transcriptase inhibitor-based regimens.^47^ We found two studies comparing these outcomes according to abacavir use with no significant differences between groups.^20^, ^33^ Furthermore, all health-care practitioners and research assistants (including RCTs) were unmasked to the antiretroviral regimen used, which might have affected the reporting of minor or moderate adverse events. Mortality was not reported systematically—only one study specified that no deaths occurred^20^—which could suggest an overestimation of our pooled mortality rate results. Additionally, the non-comparative or retrospective study design of most studies meant that most were rated to have moderate or high risk of bias, which limits our interpretation and conclusions. Finally, publication bias was reduced by the addition of grey literature in our search strategy and by discussing and sharing experiences with our paediatric HIV research network. Although our search strategy focused on articles published from January, 2009, we believe this cutoff was appropriate given that abacavir was first approved for paediatric patients in the USA in December, 2008,^48^ and was included in WHO paediatric ART recommendations in 2010.^49^ Therefore, our meta-analysis contains all studies that implemented abacavir-based regimens in line with official recommendations for dosing and clinical management.

This comprehensive systematic review summarises evidence from the past 13 years on the safety and efficacy of abacavir-based regimens in infants, children, and adolescents and aligns with the conclusions made in previous systematic reviews.^16^, ^17^ Systematic reviews conducted in adults highlighted more serious toxic effects, such as cardiovascular diseases;^10^ however, our findings support the recommendation to use abacavir as the preferred first-line regimen for infants and children. Specific data on adolescents are insufficient to provide recommendations for this age group. Future individual-level meta-analyses should further assess the efficacy of abacavir as nucleoside reverse transcriptase inhibitor backbone, especially when combined with newly introduced integrase strand transfer inhibitors. To conclude, we advocate for further research in infants, children, and adolescents living with HIV to monitor and assess ART toxicity and safety, improve ART tolerance and adherence, and improve the quality of life for this population.

## Data sharing

All data are included in the manuscript and appendix.

## Declaration of interests

CLT received consultancy fees from WHO and the International AIDS Society. JO received consultancy fees from WHO to develop this meta-analysis. VL received a consultancy contract for the University of Toulouse from WHO to develop and conduct this Review. All other authors declare no competing interests.
