# Supplementary material for: Safety and efficacy of abacavir for treating infants, children, and adolescents living with HIV: a systematic review and meta-analysis
Source: Lancet Child Adolesc Health. 2022 Oct;6(10):692–704. doi: 10.1016/S2352-4642(22)00213-9 (PMC9474298; doi:10.1016/S2352-4642(22)00213-9)
Supplement: Supplementary appendix [file mmc1.pdf]

THE LANCET
Child & Adolescent Health

Supplementary appendix

This appendix formed part of the original submission and has been peer reviewed. We post it as supplied by the authors.

Supplement to: Jesson J, Saint-Lary L, Dassi Tchoupa Revegue MH, et al. Safety and efficacy of abacavir for treating infants, children, and adolescents living with HIV: a systematic review and meta-analysis. *Lancet Child Adolesc Health* 2022; published online Sept 1. https://doi.org/10.1016/S2352-4642(22)00213-9.

SUPPLEMENTARY MATERIAL:

Table S1a: Full search terms strategy –January 2009 to October 2020

#	Searches Web of Science	Results
1	HIV	266797
2	HIV Infections*	135638
3	(HIV or HIV1 or HIV-1 or HIV2 or HIV-2 or human immun?deficiency virus* or human immun? deficiency virus*)	284775
4	(AIDS or acquired immun? deficiency syndrome* or acquired immun?deficiency syndrome*)	651292
5	#1 or #2 or #3 or #4	860187
6	abacavir	2653
7	Abacavir or ABC	36517
8	(abacavir OR 1592U89 OR abacavir sulfate OR Ziagen OR abacavir succinate)	2666
9	#6 or #7 or #8	36530
10	(adolescen* or babies or baby or boy? or boyfriend or boyhood or girlfriend or girlhood or child* or girl? or infan* or juvenil* or kid? or minors or minors* or neonat* or neo-nat* or newborn* or new-born* or paediatric* or peadiatric* or pediatric* or perinat* or preschool* or puber* or pubescen* or school* or teen* or toddler? or underage? or under-age? or youth* or young)	4301049
11	(pediatric* or paediatric* or infan* or child* or adolescen* or young)	3435321
12	#10 or #11	4301049
13	#5 and #9 and #12	667
14	(Case report* or case stud*)	2616403
15	#13 not #14	574
16	#15 has an abstract	540
17	#16 remove duplicates	538

#	Search Cochrane Library (CENTRAL)	Results
1	MeSH descriptor: [HIV] explode all trees	3034
2	MeSH descriptor: [HIV Infections] explode all trees	12403
3	((HIV or HIV1 or HIV-1 or HIV2 or HIV-2 or human immun?deficiency virus* or human immun? deficiency virus*))	43516
4	(AIDS or acquired immun? deficiency syndrome* or acquired immun?deficiency syndrome*)	17178
5	(#1-#4)	48643
6	abacavir	850
7	Abacavir OR ABC	3176
8	(abacavir OR 1592U89 OR abacavir sulfate OR Ziagen OR abacavir succinate)	853
9	(#5-#9)	3179
10	pediatric* or paediatric* or infan* or child* or adolescen* or young	350194
11	(adolescen* or babies or baby or boy? or boyfriend or boyhood or girlfriend or girlhood or child* or girl? or infan* or juvenil* or kid? or minors or minors* or neonat* or neo-nat* or newborn* or new-born* or paediatric* or peadiatric* or pediatric* or perinat* or preschool* or puber* or pubescen* or school* or teen* or toddler? or underage? or under-age? or youth* or young)	438773
12	(6-#23)	438773
13	#5 AND #9 AND #12	311
14	#13 with Publication Year from 2009 to 2020, in Trials	187
15	#13 in Cochrane Reviews	48
16	#14 or #15	235
17	#16 remove duplicates	228

#	Searches Embase	Results
1	exp HIV/	196838
2	exp HIV Infections/	379074
3	(HIV or HIV1 or HIV-1 or HIV2 or HIV-2 or human immun?deficiency virus* or human immun? deficiency virus*).mp.	505845
4	(AIDS or acquired immun? deficiency syndrome* or acquired immun?deficiency syndrome*).mp.	244083
5	or/1-4	606905
6	Abacavir/	11228
7	(abacavir OR 1592U89 OR abacavir sulfate OR Ziagen OR abacavir succinate).mp.	13420
8	Or/6-7	13420
9	(adolescen* or babies or baby or boy? or boyfriend or boyhood or girlfriend or girlhood or child* or girl? or infan* or juvenil* or kid? or minors or minors* or neonat* or neo-nat* or newborn* or new-born* or paediatric* or peadiatric* or pediatric* or perinat* or preschool* or puber* or pubescen* or school* or teen* or toddler? or underage? or under-age? or youth* or young).ti,ab,kw.	3616301
10	(pediatric* or paediatric* or infan* or child* or adolescen* or young).jn,jw.	803332
11	or/9-10	3796467
12	5 and 8 and 11	1482
13	(Case report* or case stud*).mp.	2733149
14	12 not 13	1304
15	limit 14 to yr=”2009 – Current”	843
16	Limit 15 to abstracts	795
17	Remove duplicates from 16	790

<u>Table S1b:</u> Update of the time period of the search terms strategy - October 2020 to May 2022

#	Searches Web of science	Results
1	HIV	30926
2	HIV Infections*	13316
3	(HIV or HIV1 or HIV-1 or HIV2 or HIV-2 or human immun?deficiency virus* or human immun? deficiency virus*)	32175
4	(AIDS or acquired immun? deficiency syndrome* or acquired immun?deficiency syndrome*)	73973
5	#1 or #2 or #3 or #4	94589
6	abacavir	179
7	Abacavir or ABC	8692
8	(abacavir OR 1592U89 OR abacavir sulfate OR Ziagen OR abacavir succinate)	180
9	#6 or #7 or #8	8693
10	(adolescen* or babies or baby or boy? or boyfriend or boyhood or girlfriend or girlhood or child* or girl? or infan* or juvenil* or kid? or minors or minors* or neonat* or neo-nat* or newborn* or new-born* or paediatric* or peadiatric* or pediatric* or perinat* or preschool* or puber* or pubescen* or school* or teen* or toddler? or underage? or under-age? or youth* or young)	757290
11	(pediatric* or paediatric* or infan* or child* or adolescen* or young)	551936
12	#10 or #11	757290
13	#5 and #9 and #12	88
14	(Case report* or case stud*)	430778
15	#13 not #14	79
16	#15 has an abstract	79
17	#16 remove duplicates	77

#	Search Cochrane Library (CENTRAL)	Results
#1	MeSH descriptor: [HIV] explode all trees	3255
#2	MeSH descriptor: [HIV Infections] explode all trees	13532
#3	((HIV or HIV1 or HIV-1 or HIV2 or HIV-2 or human immun?deficiency virus* or human immun? deficiency virus*))	49959
#4	(AIDS or acquired immun? deficiency syndrome* or acquired immun?deficiency syndrome*)	18806
#5	#1 OR #2 OR #3 OR #4	55763
#6	abacavir	887
#7	Abacavir OR ABC	3719
#8	(abacavir OR 1592U89 OR abacavir sulfate OR Ziagen OR abacavir succinate)	890
#9	#6 OR #7 OR #8	3722
#10	pediatric* or paediatric* or infan* or child* or adolescen* or young	390218
#11	(adolescen* or babies or baby or boy? or boyfriend or boyhood or girlfriend or girlhood or child* or girl? or infan* or juvenil* or kid? or minors or minors* or neonat* or neo-nat* or newborn* or new-born* or paediatric* or peadiatric* or pediatric* or perinat* or preschool* or puber* or pubescen* or school* or teen* or toddler? or underage? or under-age? or youth* or young)	488036
#12	#10 OR #11	488036
#13	#5 AND #9 AND #12	322
#14	#13 with Cochrane Library publication date from Oct 2020 to present, in Trials	187
#15	#13 with Cochrane Library publication date from Oct 2020 to present, in Cochrane Reviews	48
#16	#14 or #15	21
#17	#16 remove duplicates	21

#	Searches Embase	Results
1	exp HIV/	18823
2	exp HIV Infections/	30258
3	(HIV or HIV1 or HIV-1 or HIV2 or HIV-2 or human immun?deficiency virus* or human immun? deficiency virus*).mp.	53829
4	(AIDS or acquired immun? deficiency syndrome* or acquired immun?deficiency syndrome*).mp.	18198
5	or/1-4	63072
6	Abacavir/	973
7	(abacavir OR 1592U89 OR abacavir sulfate OR Ziagen OR abacavir succinate).mp.	1319
8	Or/6-7	1319
9	(adolescen* or babies or baby or boy? or boyfriend or boyhood or girlfriend or girlhood or child* or girl? or infan* or juvenil* or kid? or minors or minors* or neonat* or neo-nat* or newborn* or new-born* or paediatric* or peadiatric* or pediatric* or perinat* or preschool* or puber* or pubescen* or school* or teen* or toddler? or underage? or under-age? or youth* or young).ti,ab,kw.	520953
10	(pediatric* or paediatric* or infan* or child* or adolescen* or young).jn,jw.	108466
11	or/9-10	537256
12	5 and 8 and 11	190
13	(Case report* or case stud*).mp.	340738
14	12 not 13	168
16	Limit 15 to abstracts	164
17	Remove duplicates from 16	161
18	Limit to those published from 1st of October 2020 (by hand)	123

Table S2: Grey literature search strategies

- **Clinicaltrial.gov search strategy**

In the advanced search settings (https://clinicaltrials.gov/ct2/search/advanced), the following terms were used:

**Condition or disease:**	HIV
**Study type:**	All studies
**Study results:**	All studies
**Age group:**	Child (birth-17)
**Intervention/treatment:**	Abacavir OR ABC
**Last Update Posted:**	From 01/01/2018; To 09/21/2020

All other fields are left at the default settings.

- **WHO International Clinical Trials Registry search strategy**

In the advanced search settings (https://apps.who.int/trialsearch/AdvSearch.aspx), the following terms are used:

**In the Condition:**	HIV
**In the Intervention:**	Abacavir OR ABC

Select tick box for "Search for clinical trials in children". All other settings used are default. The search results are exported into Excel and only trials which were "Last refreshed on" 2018 to Present are retained. Trials with NCT registration (i.e. from clinicaltrials.gov) are removed as the clinicaltrials.gov website is more up to date.

- **EudraCT search strategy**

In the advanced settings (https://www.clinicaltrialsregister.eu/ctr-search/search), the following terms are used:

**Search terms:**	Abacavir OR ABC
**Age range:**	Adolescent and children and infant and toddler and newborn and preterm new born infants and under 18

Click on "Advanced Search: Search tools" to define age range above.

- **Conference abstract books** are searched for "Abacavir" in the following conferences:
  - International AIDS Society Conference 2019,2020 and 2021 (Abstracts available from: 2019:http://programme.ias2019.org/Abstract; 2020: https://www.aids2020.org/wp-content/uploads/2020/09/AIDS2020_Abstracts.pdf; 2021: https://ias2021.org/wp-content/uploads/2021/07/IAS2021_Abstracts_web.pdf)
  - Conference on Retroviruses and Opportunistic Infections (CROI; 2019 [https://www.croiconference.org/croi-2019/] ; 2020 [https://www.croiconference.org/croi-2020/]; 2021 [https://www.croiconference.org/croi-2021/] ; 2022 [https://www.croiconference.org/croi-2022/])
  - International Workshop on HIV Pediatrics (2018, 2019 and 2020 abstract books available from http://www.infectiousdiseasesonline.com/abstract-book/)
  - International Conference on AIDS and STIs in Africa 2019 and 2021 (Abstract book available from: 2019: https://saafrica.org/new/wp-content/uploads/2020/02/ICASA-2019-Abstract-Book-online-version.pdf ; 2021: http://icasa2021.saafrica.org/_docs/ICASA%202021%20Programme%20Book.pdf)

<u>Table S3:</u> Reasons for exclusion – full-text articles

Authors	Title	Year	Journal	Exclusion Reasons
Prasitsuebsai, W.; Teeraananchai, S.; Singtoroj, T. et al.	Treatment Outcomes and Resistance Patterns of Children and Adolescents on Second-Line Antiretroviral Therapy in Asia	2016	Jaids-Journal of Acquired Immune Deficiency Syndromes	Outcome not presented by drug of interest
Ashraf, I.; Ashraf, S.; Mohammad, N. and Alam, M. K.	Pharmacist-strengthen adherence to antiretroviral therapy and the contributing factors among HIV-infected paediatric patients in Nigeria	2017	Bangladesh Journal of Medical Science	Outcome not presented by drug of interest
Dimock, D.; Thomas, V.; Cushing, A. et al.	Longitudinal assessment of metabolic abnormalities in adolescents and young adults with HIV-infection acquired perinatally or in early childhood	2011	Metabolism: Clinical and Experimental	Outcome not presented by drug of interest
Walker, A. S; Prendergast, A. J.; Mugyenyi, P. et al.	Mortality in the year following antiretroviral therapy initiation in HIV-infected adults and children in Uganda and Zimbabwe	2012	Clinical Infectious Diseases	Outcome not presented by drug of interest
Santiprabhob, J.; Tanchaweng, S.; Maturapat, S. et al.	Metabolic Disorders in HIV-Infected Adolescents Receiving Protease Inhibitors	2017	BioMed Research International	Outcome not presented by drug of interest
Bobat, R.; Kindra, G.; Kiepiela, P. et al.	Use of Abacavir in 30 HIV-infected Children From Durban, South Africa Report From a Pilot Study	2010	Pediatric Infectious Disease Journal	Wrong publication type
Bwakura-Dangarembizi, M.; Musiime, V.; Szubert, A. et al.	Prevalence of Lipodystrophy and Metabolic Abnormalities in HIV-infected African Children after 3 Years on First-line Antiretroviral Therapy	2015	Pediatric Infectious Disease Journal	Outcome not presented by drug of interest
Chanthong, P.; Lapphra, K.; Saihongthong, S. et al.	Echocardiography and carotid intima-media thickness among asymptomatic HIV-infected adolescents in Thailand	2014	Aids	Outcome not presented by drug of interest
Dziuban, E; DeVos, J.; Ngeno, B. et al.	High Prevalence of Abacavir-associated L74V/I Mutations in Kenyan Children Failing Antiretroviral Therapy	2017	Pediatric Infectious Disease Journal	Wrong outcome
Flynn, P.; Komar, S; Blanche, S. et al.	Efficacy and Safety of Darunavir/Ritonavir at 48 Weeks in Treatment-naive, HIV-1-infected Adolescents Results From a Phase 2 Open-label Trial (DIONE)	2014	Pediatric Infectious Disease Journal	No specific data on drug of interest
Frigati, L.; Brown, K.; Mahtab, S. et al.	Multisystem impairment in South African adolescents with Perinatally acquired HIV on antiretroviral therapy (ART)	2019	Journal of the International Aids Society	Outcome not presented by drug of interest
Frigati, L.; Jao, J.; Mahtab, S et al.	Insulin Resistance in South African Youth Living with Perinatally Acquired HIV Receiving Antiretroviral Therapy	2019	Aids Research and Human Retroviruses	Outcome not presented by drug of interest
Githinji, L. N.; Mahtab, S.; Zuhlke, L. et al.	Cardiopulmonary dysfunction in perinatally HIV-infected South African adolescents on antiretroviral therapy: baseline findings from the Cape Town Adolescent Antiretroviral Cohort	2019	Journal of the International Aids Society	Outcome not presented by drug of interest
Jacobson, D. L.; Williams, P.; Tassiopoulos, K.; Melvin, A.; Hazra, R.; Farley, J.	Clinical management and follow-up of hypercholesterolemia among perinatally hiv-infected children enrolled in the PACTG 219C study	2011	Journal of acquired immune deficiency syndromes	Outcome not presented by drug of interest
Jevtovic, D.; Salemovic, D.; Ranin, J.; Brmbolic, B.; Djurkovic-Djakovoc, O.	The prognosis of pediatric AIDS in Serbia	2009	Current Hiv Research	No specific data on drug of interest

Kityo, C.; Szubert, A. J.; Siika, A. et al.	Raltegravir-intensified initial antiretroviral therapy in advanced HIV disease in Africa: a randomised controlled trial	2018	Plos Medicine	Wrong outcome
Kyaw, N; Kumar, A.; Oo, M et al.	Long-term outcomes of second-line antiretroviral treatment in an adult and adolescent cohort in Myanmar	2017	Global Health Action	Outcome not presented by drug of interest
Lilian, R. R.; Mutasa, B.; Railton, J. et al.	A 10-year cohort analysis of routine paediatric ART data in a rural South African setting	2017	Epidemiology and Infection	Outcome not presented by drug of interest
Lumbiganon, P.; Kosalaraksa, P.; Bunupuradah, T. et al.	HIV-infected children in the Asia-Pacific region with baseline severe anemia: antiretroviral therapy and outcomes	2016	Asian Biomedicine	No specific data on drug of interest
Musiime, V.; Kasirye, P.; Naidoo-James, B. et al.	Once vs twice-daily abacavir and lamivudine in African children	2016	AIDS (London, England)	Outcome not presented by drug of interest
Sarni, R. O. S.; De Souza, F. I. S.; Battistini, T. R. B. et al.	Lipodystrophy in children and adolescents with acquired immunodeficiency syndrome and its relationship with the antiretroviral therapy employed	2009	Jornal de Pediatria	Outcome not presented by drug of interest
Szubert, A. J.; Prendergast, A. J.; Spyer, M. J. et al.	Virological response and resistance among HIV-infected children receiving long-term antiretroviral therapy without virological monitoring in Uganda and Zimbabwe: observational analyses within the randomised ARROW trial	2017	Plos Medicine	Outcome not presented by drug of interest
Tadesse, B. T.; Foster, B. A.; Kabeta, A. et al.	Hepatic and renal toxicity and associated factors among HIV-infected children on antiretroviral therapy: a prospective cohort study	2019	Hiv Medicine	Outcome not presented by drug of interest
Viljoen, E.; MacDougall, C.; Mathibe, M.; Veldman, F.; Mda, S.	Dyslipidaemia among HIV-infected children on antiretroviral therapy in Garankuwa, Pretoria	2020	South African Journal of Clinical Nutrition	Outcome not presented by drug of interest
Voronin, E. E.; Fortuny, C.; Perez-Tamarit, D. et al.	Pharmacokinetics, safety, and antiviral activity of fosamprenavir-containing twice-daily regimens in HIV-infected children 2-18 years old: Report from APV29005, a 48-week prospective, open-label, multicenter cohort study	2012	Pharmacotherapy	Wrong publication type
Wamalwa, D. C.; Lehman, D. A.; Benki-Nugent, S. et al.	Long-term virologic response and genotypic resistance mutations in HIV-1 infected kenyan children on combination antiretroviral therapy	2013	Journal of acquired immune deficiency syndromes	Outcome not presented by drug of interest
Wekesa, P.; Nyabiage, L.; Owuor, K. et al.	Towards the third 90: Factors associated with adolescent antiretroviral adherence and viral suppression	2018	Journal of the International Aids Society	Wrong publication type
Kingwara, L.; Inzaule, SC; Momanyi, L. et al.	Impact of nucleos(t)ide reverse transcriptase inhibitor resistance on dolutegravir and protease-inhibitor-based regimens in children and adolescents in Kenya	2022		Outcome not presented by drug of interest
Mbuya, W.; Mwakyula, I.; Olomi, W. et al.	Altered Lipid Profiles and Vaccine Induced-Humoral Responses in Children Living With HIV on Antiretroviral Therapy in Tanzania	2021	Frontiers in Cellular and Infection Microbiology	No specific data on drug of interest
Bossacoma Busquets, F.; Sanchez, E.; Noguera-Julian, A. et al.	Weight-adapted fixed-dose combined adult antiretroviral tablets for HIV-infected children	2021	Journal of Clinical Pharmacy and Therapeutics	No specific data on drug of interest

Dirajlal-Fargo, S.; Albar, Z.; Bowman, E. et al.	Subclinical Vascular Disease in Children with Human Immunodeficiency Virus in Uganda Is Associated with Intestinal Barrier Dysfunction	2020	Clinical Infectious Diseases	No specific data on drug of interest
Tiam, A.; MacHecano, R.; Walner, K. et al.	HIV-infected treatment-experienced children and adolescents from Sub-Saharan Africa: Clinical outcomes on thirdline antiretroviral treatment in the New Horizons drug donation programme	2020	Journal of the International AIDS Society	Outcome not presented by drug of interest
Osman, F.T. and Yizengaw M.A.	Virological failure and associated risk factors among HIV/AIDS pediatric patients at the ART clinic of jimma university medical center, southwest Ethiopia	2020	Open AIDS Journal	Outcome not presented by drug of interest

<u>Figure S3</u>: Summary of risk of bias assessment stratified by study design: a) randomised controlled trials (Cochrane Risk of Bias tool 2.0), b) single-arm trials (National Institute of Health quality assessment tool), c) observational studies (Clinical Advances through Research and Information Translation tool).

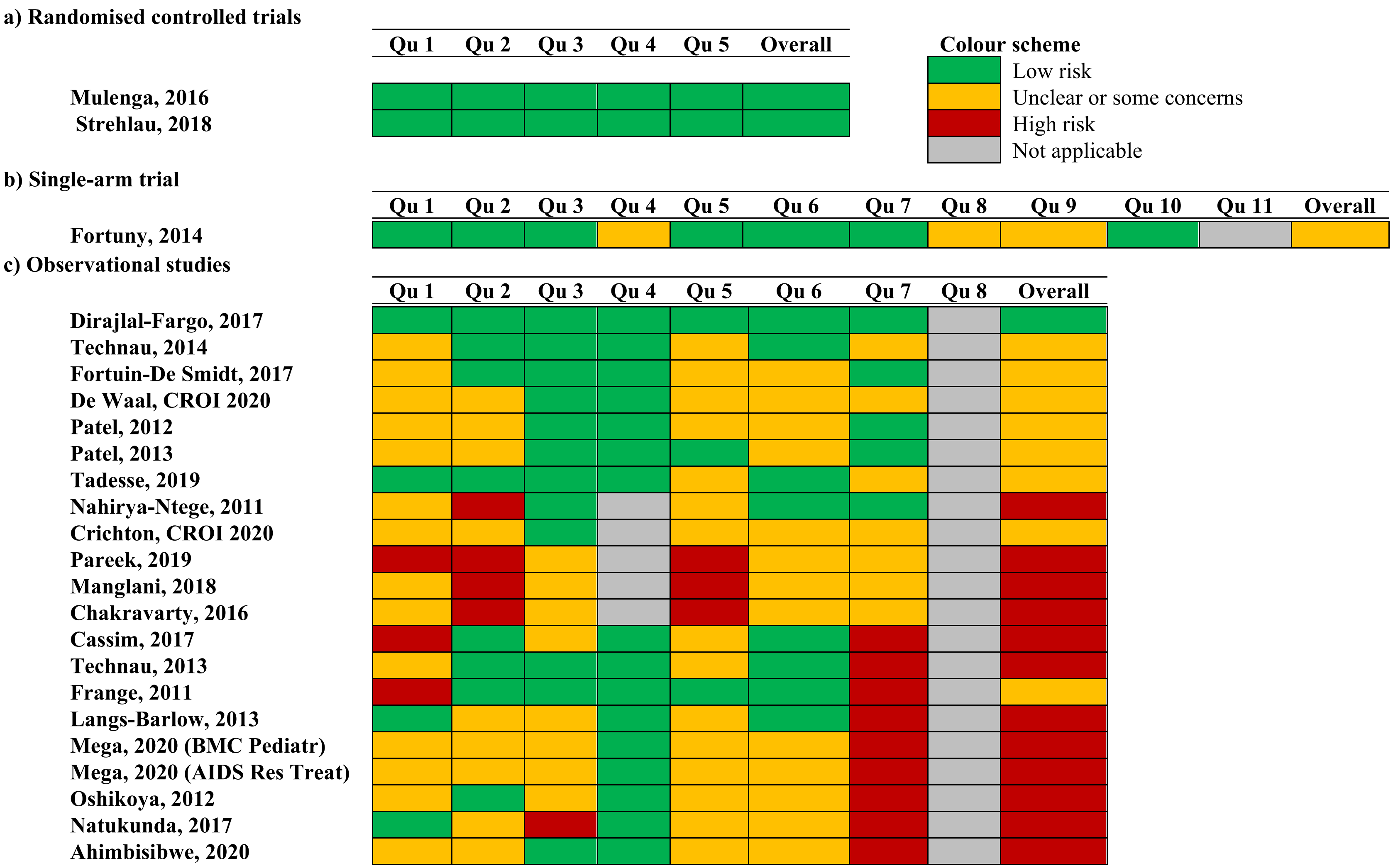

Legend a)
Domain 1: Risk of bias from the randomisation process
Domain 2: Risk of bias due to deviations from the intended interventions
Domain 3: Missing outcome data

Domain 4: Risk of bias in measurement of the outcome
Domain 5: Risk of bias in selection of the reported result

Legend b)

Qu 1: Was the study question or objective clearly stated?
Qu 2: Were eligibility/selection criteria for the study population prespecified and clearly described?
Qu 3: Were the participants in the study representative of those who would be eligible for the intervention in the general or clinical population of interest?
Qu 4: Were all eligible participants that met the prespecified entry criteria enrolled?
Qu 5: Was the sample size sufficiently large to provide confidence in the findings?
Qu 6: Was the intervention clearly described and delivered consistently across the study population?
Qu 7: Were the outcome measures prespecified, clearly defined, valid, reliable, and assessed consistently across all study participants?
Qu 8: Were the people assessing the outcomes blinded to the participants' interventions?
Qu 9: Was the loss to follow-up after baseline 20% or less? Were those lost to follow-up accounted for in the analysis?
Qu 10: Did the statistical methods examine changes in outcomes measures from before to after the intervention? Were statistical tests done that provided p values for the pre-to-post changes?
Qu 11: Were outcome measures of interest taken at baseline and multiple times after the intervention?
Qu 12: If the intervention was conducted at a group level did the statistical analysis take into account the use of individual-level data to determine effects at the group level?

Legend c)

Qu 1: Was the study population selected in an appropriate way?
Qu 2: Can we be confident in the assessment of exposure?
Qu 3: Can we be confident that the outcome of interest was not present at start of study?
Qu 4: For comparative studies: did the study match exposed and unexposed for all variables that are associated with the outcome of interest or did the statistical analysis adjust for these prognostic variables?
Qu 5: Can we be confident in the assessment of the presence or absence of prognostic factors?
Qu 6: Can we be confident in the assessment of outcome?
Qu 7: Was the follow-up of cohorts adequate? (Both duration and completeness of follow-up, i.e are all subjects accounted for?)
Qu 8: For comparative studies: were co-interventions similar between groups?

Table S3: Efficacy outcomes (viral load) in children and adolescents treated with ABC-containing drug regimen, systematic review 2009-2020:

First author, year publication, age groups	Control group	Viral Load <400 copies/mL		Viral Load <50 copies/mL		Other viral load outcomes	
		Abacavir	Control	Abacavir	Control	Abacavir	Control
**Mulenga, 2016**<br><br>**Children**	Stavudine and Zidovudine	ART-naive:<br>48 weeks=95 (81%)<br>96 weeks=91 (82%)<br><br>previously experienced on ART:<br>48 weeks=33 (97%)<br>96 weeks=91 (97%)	Stavudine:<br>ART-naive:<br>48 weeks=98 (85%) / 96 weeks=80 (75%)<br>previously experienced on ART:<br>48 weeks=31 (97%); 96 weeks=31 (97%)<br>Zidovudine:<br>ART-naive:<br>48 weeks=81 (80%); 96 weeks=78 (76%)<br>previously experienced on ART:<br>48 weeks=43 (96%); 96 weeks=45 (100%)	NA	NA	NA	NA
**Dirajlal-Fargo, 2017 Children**	Stavudine and Zidovudine	63%	Stavudine: 57%<br>Zidovudine: 66%	NA	NA	NA	NA
**Strehlau, 2018 Children**	Stavudine	NA	NA	NA	NA	Probability of viral rebound >50 copies/mL=0.240<br>Probability confirmed viral failure >1000 copies/mL=0.019	Probability of viral rebound >50 copies/mL=0.295, p =0.233<br>Probability confirmed viral failure >1000 copies/mL=0.033, p=0.608
**Fortuny, 2014 Children & Adolescents**	NA	n=84/109, 77%	NA	NA	NA	NA	NA
**Technau, 2014**<br><br>**Infants & Children**	Stavudine	At 6 months:<br>ritonavir-boosted Lopinavir based: 54%<br>Efavirenz based: 78%<br><br>At 12 months:<br>ritonavir-boosted	At 6 months:<br>ritonavir-boosted Lopinavir based: 70%, p<0.001<br>Efavirenz based: 86%, p<0.001<br>OR Stavudine versus Abacavir, ritonavir-boosted Lopinavir based: 0.49 (0.40-0.60)	NA	NA	NA	NA

		Lopinavir based: 62%<br>Efavirenz based: 75%	aOR Stavudine versus Abacavir, Efavirenz based: 0.56 (0.43-0.72)<br><br><u>At 12 months</u><br>ritonavir-boosted Lopinavir based: 77%, p<0.001<br>Efavirenz based: 84%, p<0.001<br>OR Stavudine versus Abacavir, ritonavir-boosted Lopinavir based: 0.52 (0.39-0.69)<br>aOR Stavudine versus Abacavir, Efavirenz based: 0.56 (0.36-0.86)				
**Fortuin-De Smidt, 2017**<br><br>**Children**	Most common: Lamivudine -Stavudine + either Efavirenz or ritonavir-boosted Lopinavir	NA	NA	NA	NA	Discontinuations due to virological, immunological or clinical failure: 30/102 (5%)	Discontinuations due to virological, immunological or clinical failure: 207 (34%)
**De Waal, CROI 2020 Infants**	Zidovudine	At 6 months: 50%<br>At 12 months: 57%	At 6 months: 66%, OR 0.6 (95%CI 0.3 to 1.2)<br>At 12 months: 56%, OR 1.4 (95%CI 0.8 to 2.5)	At 6 months: 23%<br>At 12 months: 24%	At 6 months: 17%<br>At 12 months: 15%	/	/
**Tadesse, 2019 Children & Adolescents**	Zidovudine and Tenofovir disoproxil fumarate	NA	NA	NA	NA	Achieving undetectable viremia (<150 copies per mL) during follow-up: n=34, 92%<br>Virological rebound for those who achieved virological suppression: n=15, 42%	Achieving undetectable viremia (<150 copies per mL) during follow-up:<br>Zidovudine: n=13, 87%<br>TDF: n=47, 81%<br>Virological rebound for those who achieved virological suppression:<br>Zidovudine: n=5, 14%<br>TDF: n=16, 44%
**Crichton, CROI 2020 Infants**	NA	At 6 months: 70%<br>At 12 months: 77%<br>No diff by regimen	NA	NA	NA	NA	NA
**Cassim, 2017 Infants**	Stavudine	At 6 months: n=30 54.5%)<br>At 12 months: n=34 (66.7%)	At 6 months: n=67 (67.0%), p=0.13<br>At 12 months: n=73 (71.6%), p=0.53	NA	NA	NA	NA
**Technau, 2013**	Stavudine, with either Efavirenz or ritonavir-	<u>For Abacavir/Lamivudine overall:</u><br>At 6 months: 106/194	<u>For Stavudine/Lamivudine overall:</u><br>At 6 months: 686/831 (83%)<br>At 12 months: 891/1057 (84%), p<0.0001	NA	NA	NA	NA

**Infants & Children**	boosted Lopinavir	(55%)<br>At 12 months: 121/198 (61%)<br>For Abacavir/Lamivudine/ritonavir-boosted Lopinavir:<br>At 6 months: 35/88 (64%)<br>At 12 months: 41/81 (51%)<br>For Abacavir/Lamivudine/Efavirenz:<br>At 6 months: 71/106 (67%)<br>At 12 months: 80/117 (68%)	For Stavudine/Lamivudine/ritonavir-boosted Lopinavir:<br>At 6 months: 249/351 (71%)<br>At 12 months: 308/398 (77%)<br>For Stavudine/Lamivudine/Efavirenz:<br>At 6 months: 437/480 (91%)<br>At 12 months: 583/659 (88%)<br><br>aOR Failure to reach <400 copies/mL, Stavudine versus Abacavir:<br>ritonavir-boosted Lopinavir based: 0.36 (0.18–0.72)<br>Efavirenz based: 0.31 (0.16–0.60)				
**Frange, 2011**<br><br>**Children**	ritonavir-boosted Lopinavir based with Lamivudine, Zidovudine or Stavudine	NA	NA	Failure to achieve a VL < 50 copies/mL by 12 months:<br>Abacavir versus no: 52.6% versus 12.5%, OR=7.8 (1.7-35.1)	Zidovudine versus no: 23.1% versus 41.2%, OR=0.4 (0.1-1.6)<br>Lamivudine versus no: 27.8% versus 42.9%, OR=0.5 (0.1-2.7)<br>Stavudine versus no: 0.0% versus 34.2%	NA	NA
**Ahimbisibwe, 2020**<br><br>**Infants, Chidren & Adolescents**	Zidovudine and Tenofovir disoproxil fumarate	NA	NA	NA	NA	VL <1000 copies/mL: n=17, 53.1%	VL <1000 copies/mL:<br>Zidovudine : n=235, 37.3%,<br>Tenofovir disoproxil fumarate: N=39 , 27.5%,<br>aOR (on age, sex, duration and prior exposure to ART, WHO staging):<br>Zidovudine versus Abacavir=1.9 (0.9-3.9)<br>Tenofovir disoproxil fumarate versus Abacavir=3.1 (1.4-6.8)

OR= Odds Ratio, RCT=Randomised Clinical Trial, VL=VL.. NA = outcomes not reported or no comparative group
Age groups: Infants = 0-12 months, Children = 1-10 years, Adolescents = 10-19 years

Table S4: Efficacy outcomes (CD4) in children and adolescents treated with ABC-containing drug regimen, systematic review 2009-2020

First author, year publication, age groups	Control group	CD4 outcomes (cell count or %)	
		ABC	Control
**Strehlau, 2018 Children**	Stavudine	CD4% at 32 wks (Mean ± SD, n=100)= 36.0 ± 7.3<br>CD4% at 56 wks (Mean ± SD, n=102)= 36.5 ± 6.7	CD4% at 32 wks (Mean ± SD, n=99)= 35.2 ± 7.8, p-value=0.441<br>CD4% at 56 wks (Mean ± SD, n=96)= 36.4 ± 6.9, p-value=0.950
**Pareek, 2019 Children & Adolescents**	NA	N=48, Mean (SD) CD4 count improved from 648 (463) /mm3 to 790 (381) /mm3 after 1 year (P=0.006).<br>Immunological failure = 7 (14.5%)	NA
**Cassim, 2017 Infants**	Stavudine	N=57, CD4>25%:<br>At 6 mo: n=39 (73.6%)<br>At 12 mo: n=40 (78.4%)	N=114, CD4>25%:<br>At 6 mo: n=74 (73.4%), p=0.97<br>At 12 mo: n=85 (84.2%), p=0.62
**Mega, 2020 Children**	Zidovudine	N=87, Mean CD4 count change at 6, 12 and 18 mo (cells/mm3): 55.3, 66.3, 87.7	N=92, Mean CD4 count change at 6, 12 and 18 mo (cells/mm3): 41.6, 63.1, 65.1<br>CD4+ count gain per 6 mo, ZDV vs ABC: adjusted β = 20.51, 95% CI [6.37–34.65], p=0.004

NA= outcomes not reported or no comparative group.
Age groups: Infants = 0-12 months, Children = 1-10 years, Adolescents = 10-19 years
